# Supplementary material for: Impurity contribution to ultraviolet absorption of saturated fatty acids
Source: Sci Adv. 2023 Sep 20;9(38):eadj6438. doi: 10.1126/sciadv.adj6438 (PMC10511181; doi:10.1126/sciadv.adj6438)
Supplement: Supplementary file 1 — Figs. S1 to S15 Tables S1 to S7 [file sciadv.adj6438_sm.pdf]

Science Advances
AAAS

Supplementary Materials for

Impurity contribution to ultraviolet absorption of saturated fatty acids

Shota Saito *et al.*

Corresponding author: Tetsuya Hama, hamatetsuya@g.ecc.u-tokyo.ac.jp

Sci. Adv. **9**, eadj6438 (2023)
DOI: 10.1126/sciadv.adj6438

This PDF file includes:

Figs. S1 to S15
Tables S1 to S7

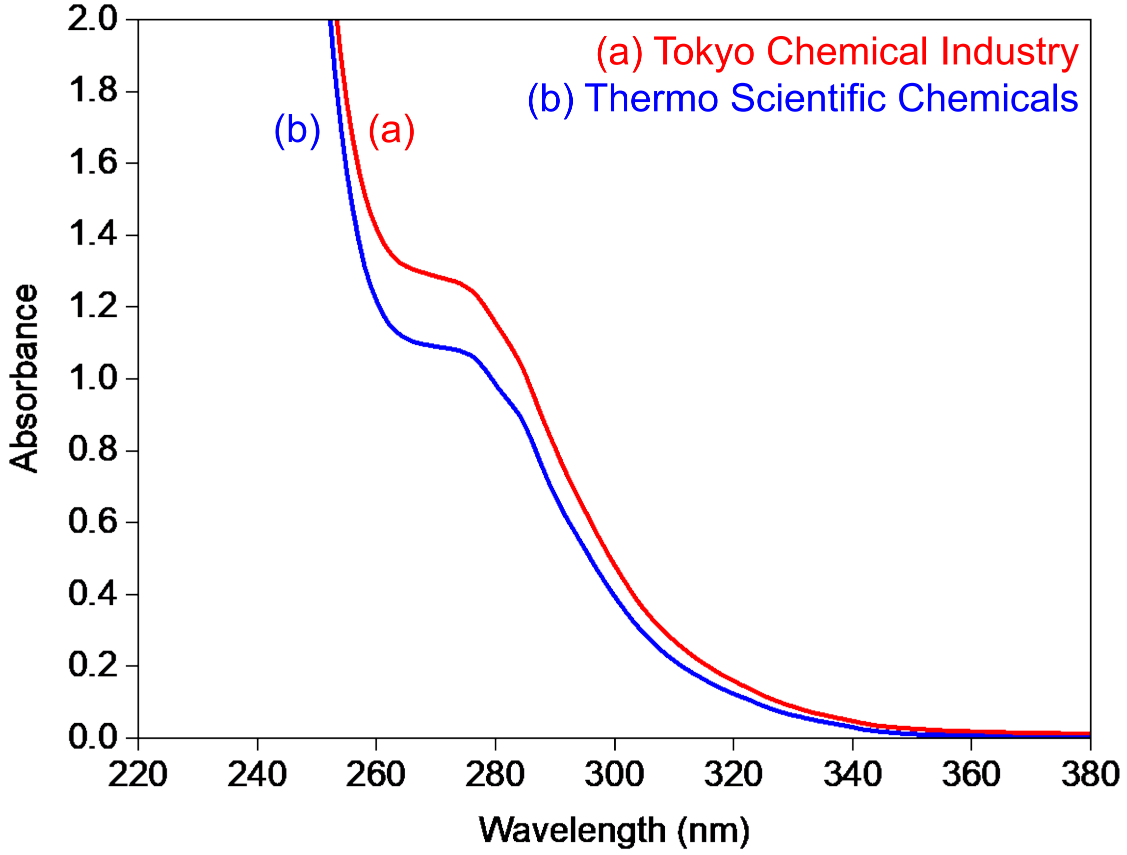

Fig. S1.

UV absorption spectra of nonanoic acid from two sources before purification by recrystallization. (a) Tokyo Chemical Industry (purity > 98.0%) and (b) Thermo Scientific Chemicals (purity 97%). To measure large absorbance values, purified water was used as a transparent reference in the background measurement. The optical path lengths of the quartz cuvettes were 10 mm for both background and sample measurements.

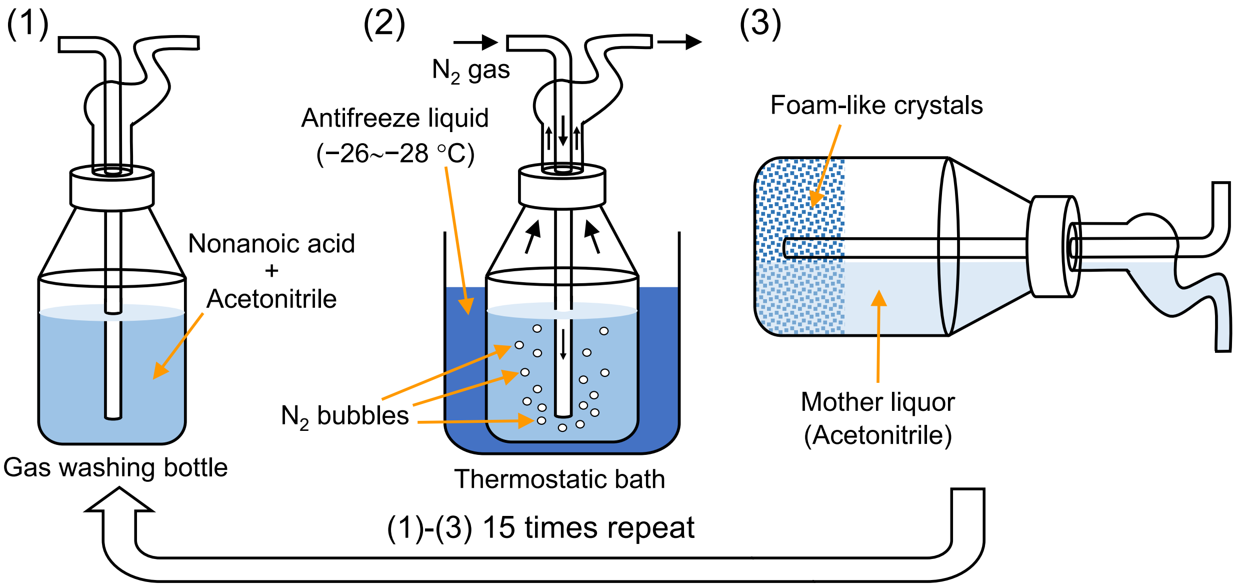

Fig. S2.
Recrystallization system.

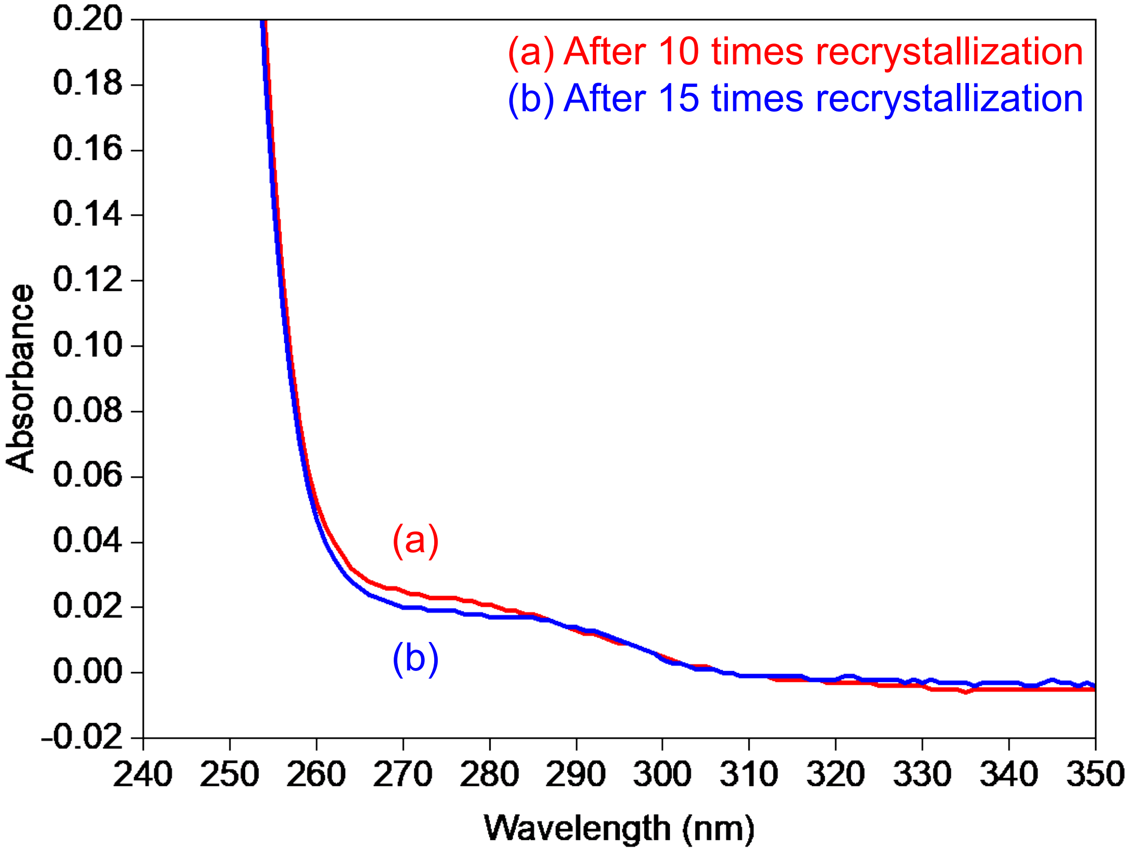

Fig. S3.

UV absorption spectra of purified nonanoic acid recrystallized (a) 10 and (b) 15 times. The optical path lengths of the quartz cuvettes for background and sample measurements were L^b = 10 mm and L^s = 20 mm; i.e., $L = L^s - L^b = 10$ mm. Both cuvettes contained identical nonanoic acid samples.

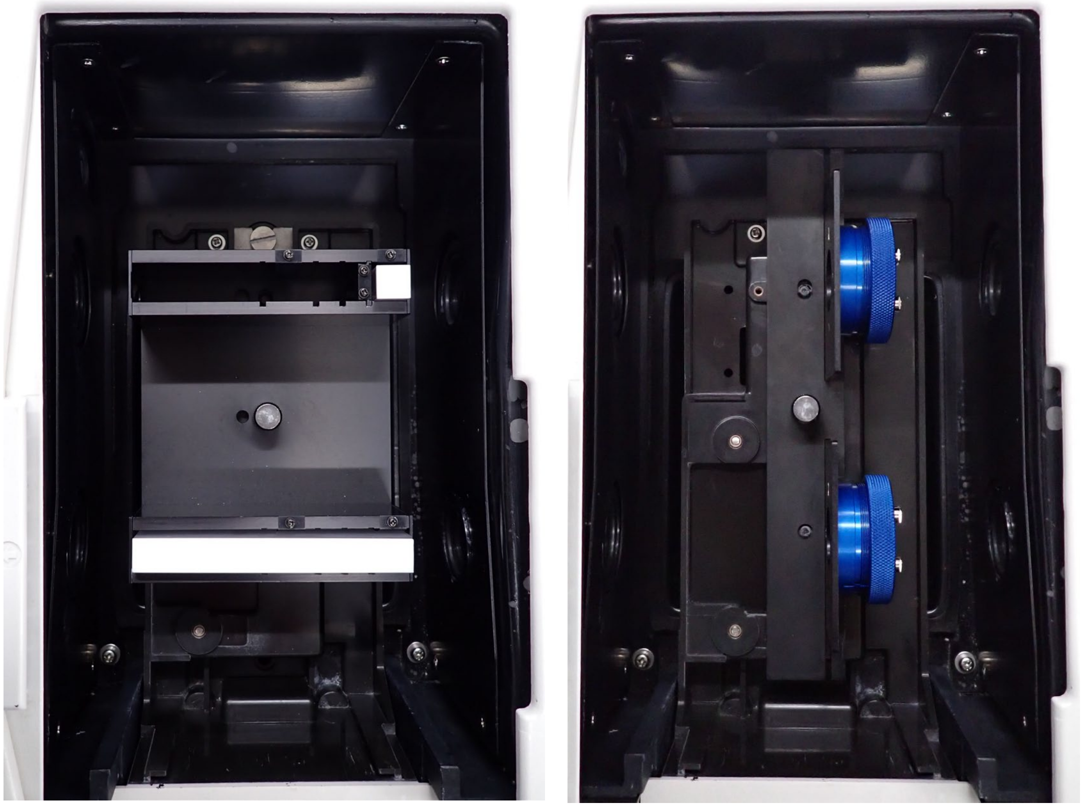

Fig. S4.

Photographs of UV absorption measurements. (Left) UV absorption measurements using rectangular quartz cuvettes (with Teflon lids) having L^b = 10 mm (upper cuvette) and L^s = 100 mm (lower cuvette) and thus $L = L^s - L^b = 90$ mm. (Right) UV absorption measurements using two demountable liquid cells.

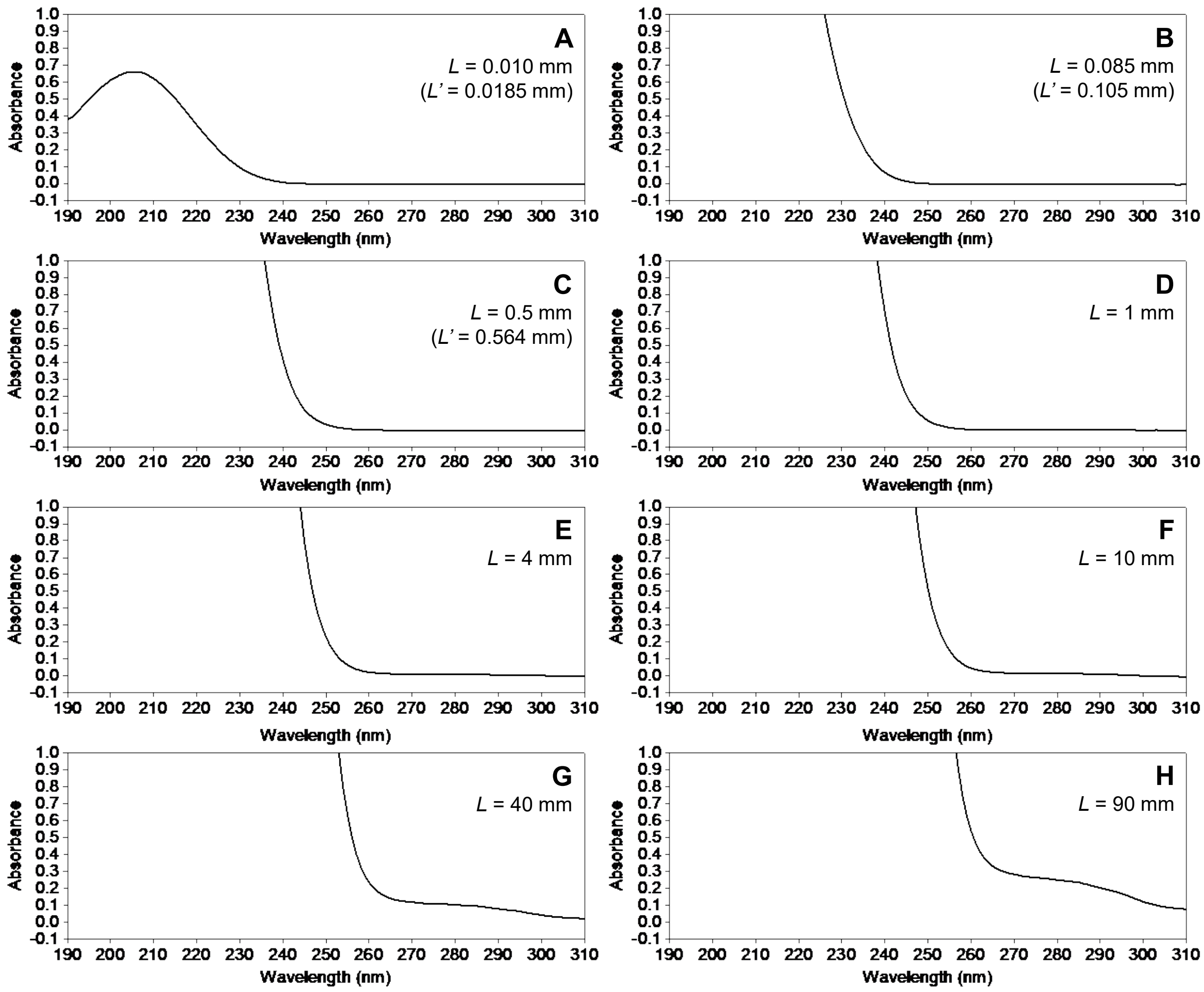

Fig. S5.

UV absorption spectra of nonanoic acid purified by recrystallization 15 times. Optical path lengths (L) were (A) 0.010 mm, (B) 0.085 mm, (C) 0.5 mm, (D) 1 mm, (E) 4 mm, (F) 10 mm, (G) 40 mm, and (H) 90 mm. The actual optical path lengths (L') are also shown for the spectra (A), (B), and (C) measured by a liquid film method with demountable liquid cells. Table S1 summarizes the measurement conditions.

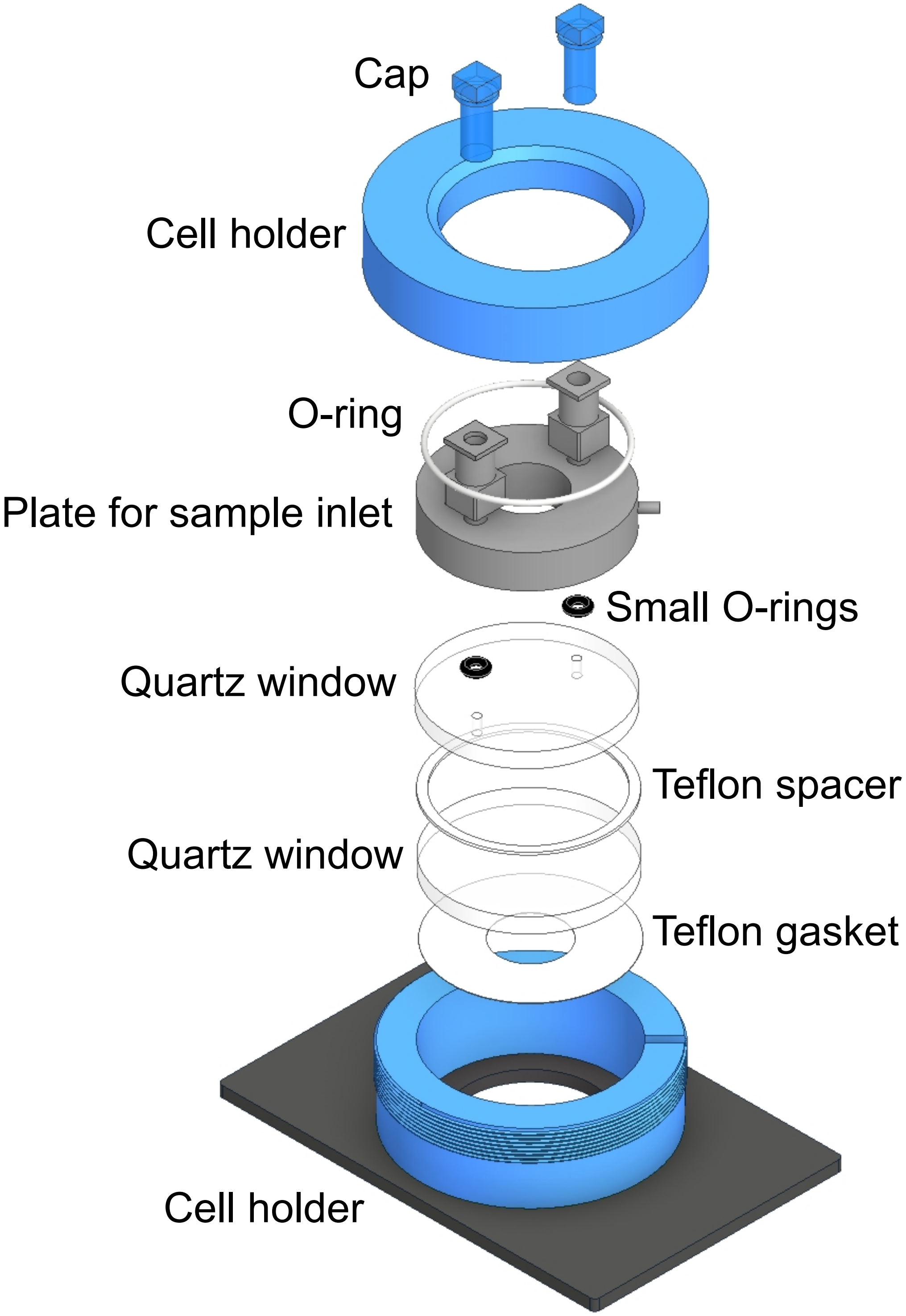

Fig. S6.
A demountable liquid cell.

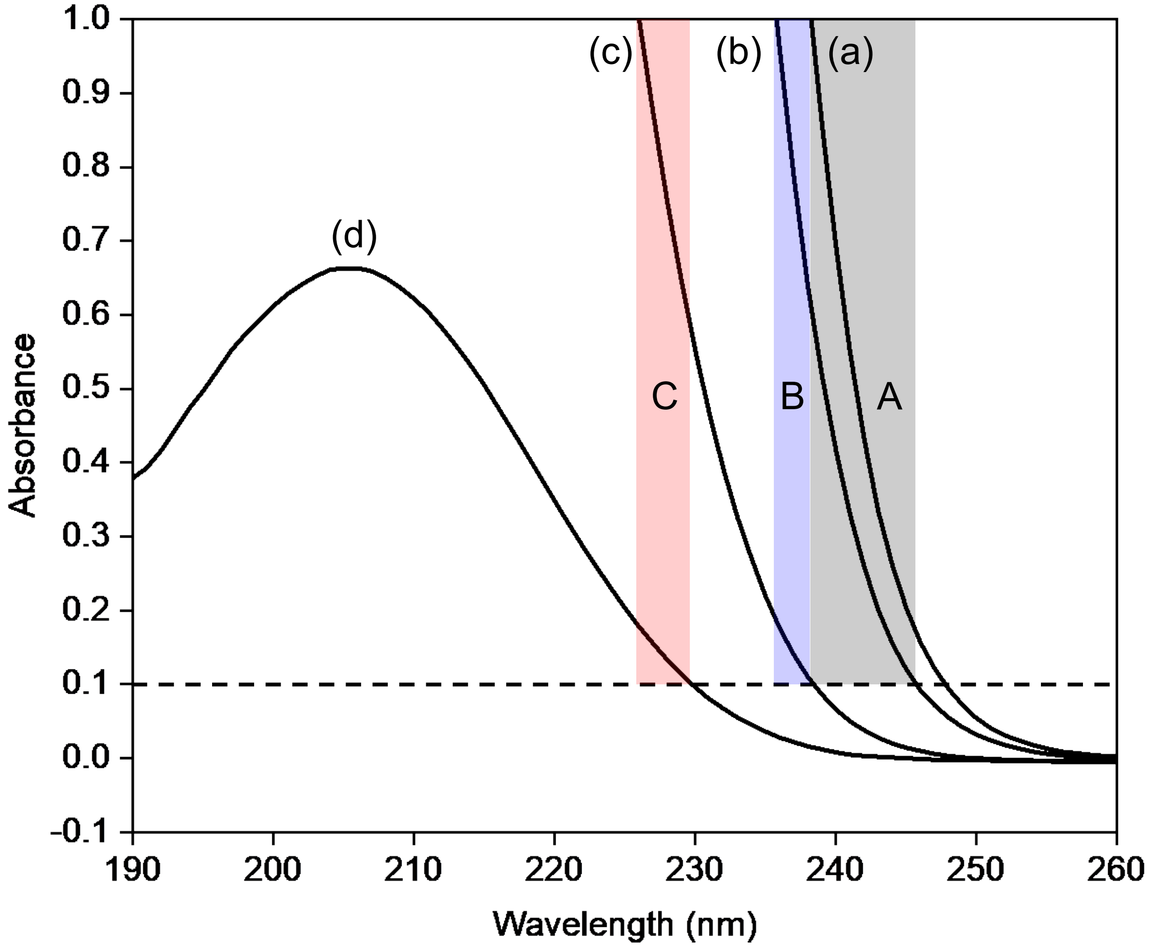

Fig. S7.

UV absorption spectra of nonanoic acid purified by recrystallization 15 times. Optical path lengths (*L*) were (a) 1 mm, (b) 0.5 mm, (c) 0.085 mm, and (d) 0.010 mm. The wavelength regions A, B, and C represent the overlap of the two spectra (a) and (b), (b) and (c), and (c) and (d) in the spectral range with the absorbance (*A*) values of $0.1 \leq A \leq 1$, respectively. These three wavelength regions were used to calculate the actual optical path lengths (*L*') for the spectra (b), (c), and (d) measured by a liquid film method with demountable liquid cells. Table S1 summarizes the measurement conditions.

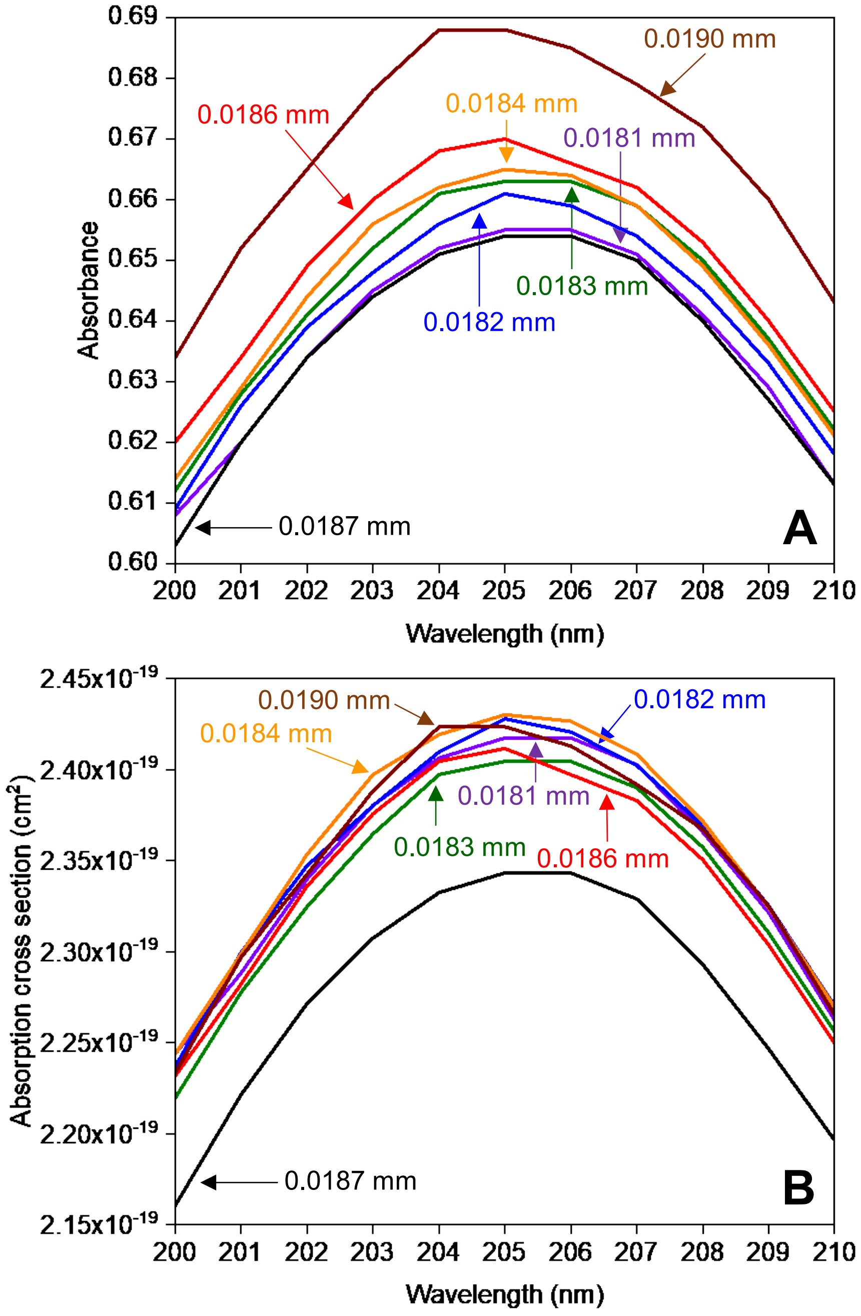

Fig. S8.

Seven independent UV absorption measurements of nonanoic acid purified by recrystallization 15 times using two demountable liquid cells. (A) UV absorption spectra of nonanoic acid. Optical path length (L) was set to 0.010 mm. The actual optical path length (L') is also shown for each spectrum. Table S1 summarizes the measurement conditions. (B) The corresponding calculated absorption cross sections.

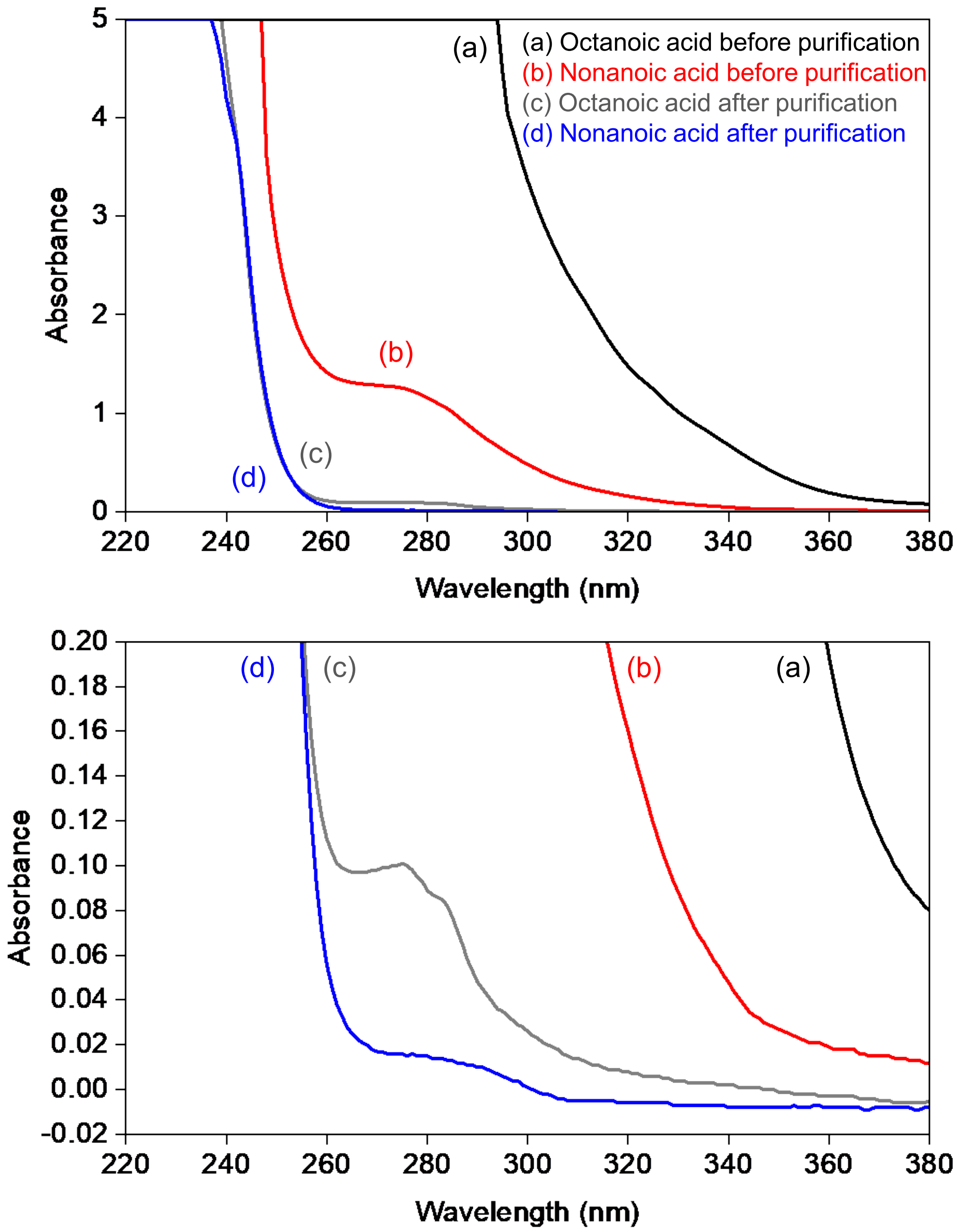

Fig. S9.

UV absorption spectra of octanoic acid and nonanoic acid. (a) octanoic acid and (b) nonanoic acid before purification; (c) octanoic acid and (d) nonanoic acid after purification by recrystallization 15 times. The lower plot is an enlargement of the upper. To measure large absorbance values, purified water was used as a transparent reference in the background measurement. The optical path length of the quartz cuvettes was 10 mm for both the background and sample measurements.

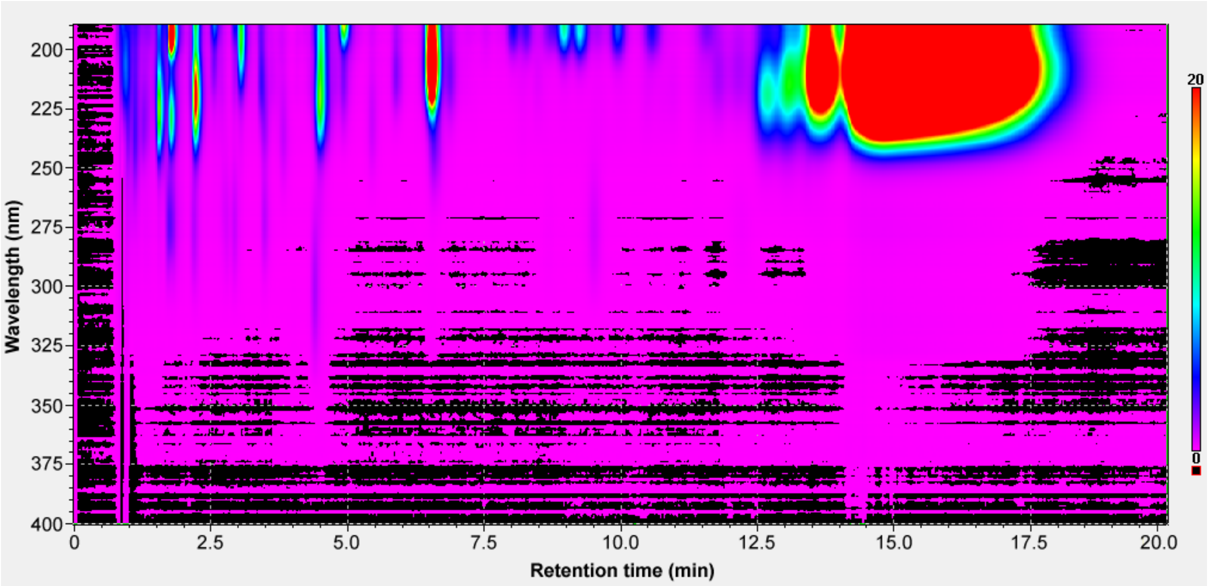

Fig. S10.
HPLC chromatogram of commercial nonanoic acid (TCI). The scale bar indicates the coloring pattern: <0 mAU (black), 0–20 mAU (violet to red). The largest peak (from 14 min) was predominantly from nonanoic acid. The second largest peak (13.5 min) was from a compound assumed to be 2-methyloctanoic acid [2-MOA, $CH_3(CH_2)_4CH(CH_3)COOH$]. This spectrum clarifies that the sample contained many impurities that show peaks at >250 nm.

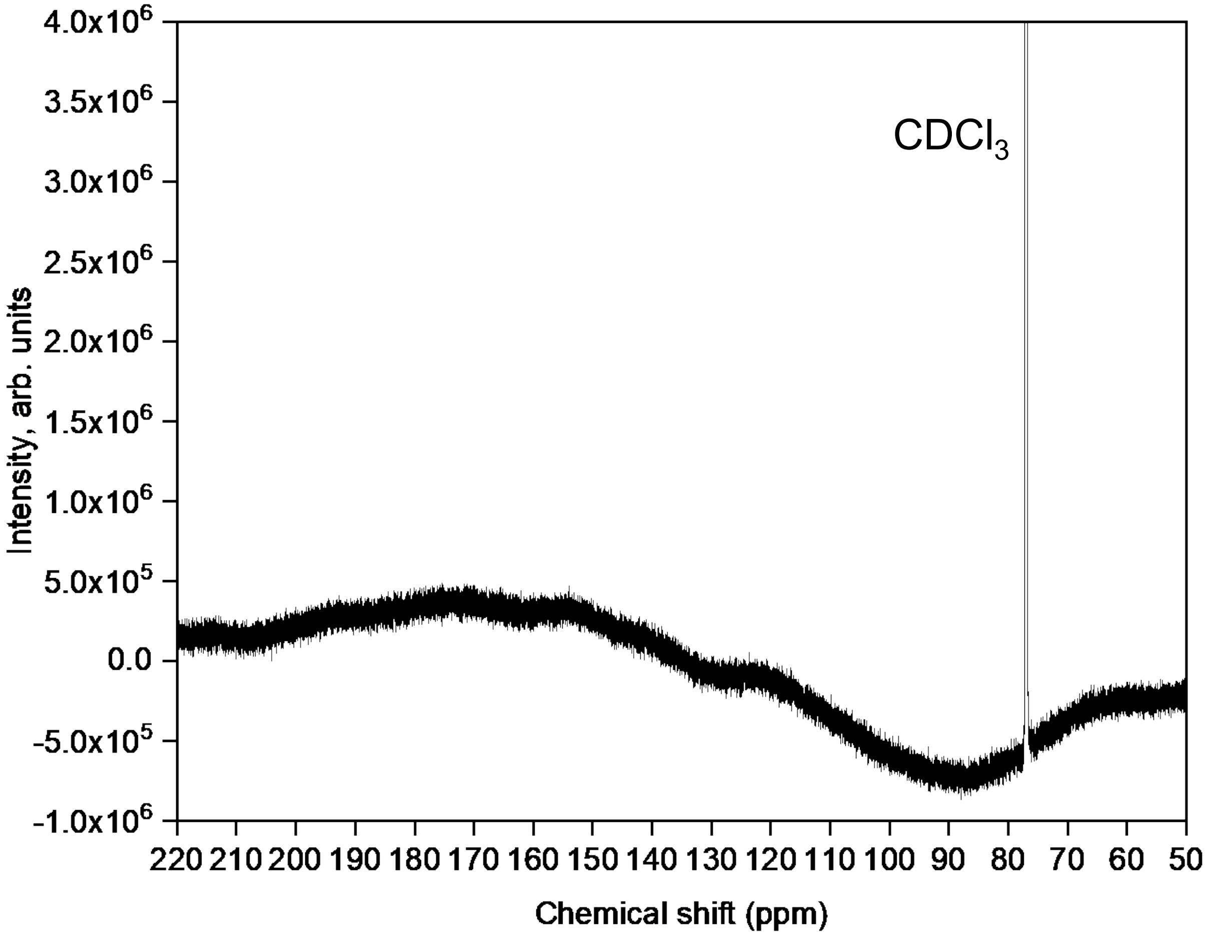

Fig. S11.
^{13}C NMR spectra of deuterated chloroform ($CDCl_3$) showing no clear impurity peak.

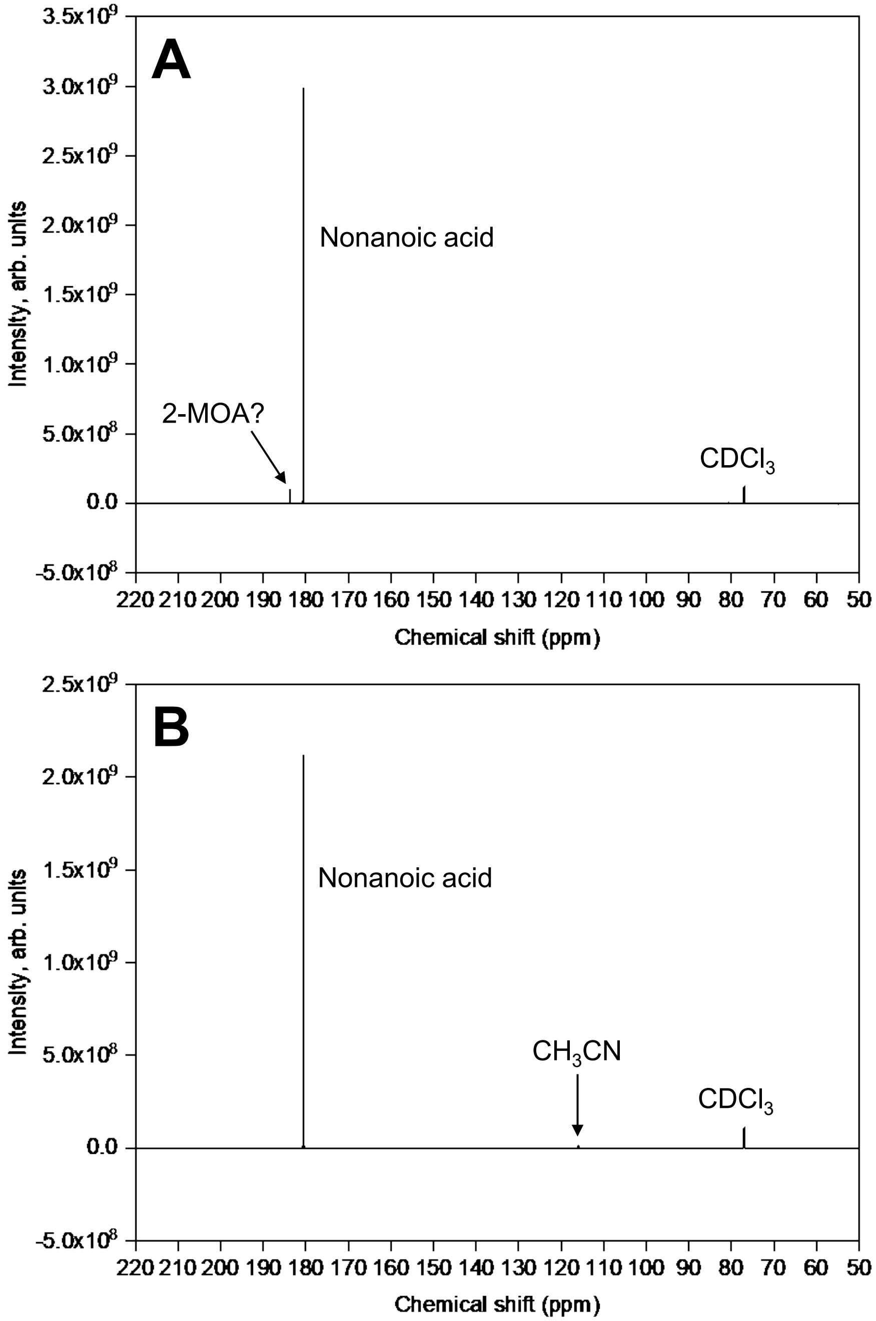

Fig. S12.
^{13}C NMR spectra of 50% deuterated chloroform ($CDCl_3$) solutions of commercial nonanoic acid (TCI) (A) before and (B) after recrystallization. 2-MOA represents 2-methyloctanoic acid [$CH_3(CH_2)_4CH(CH_3)COOH$] as a candidate for the most abundant impurity.

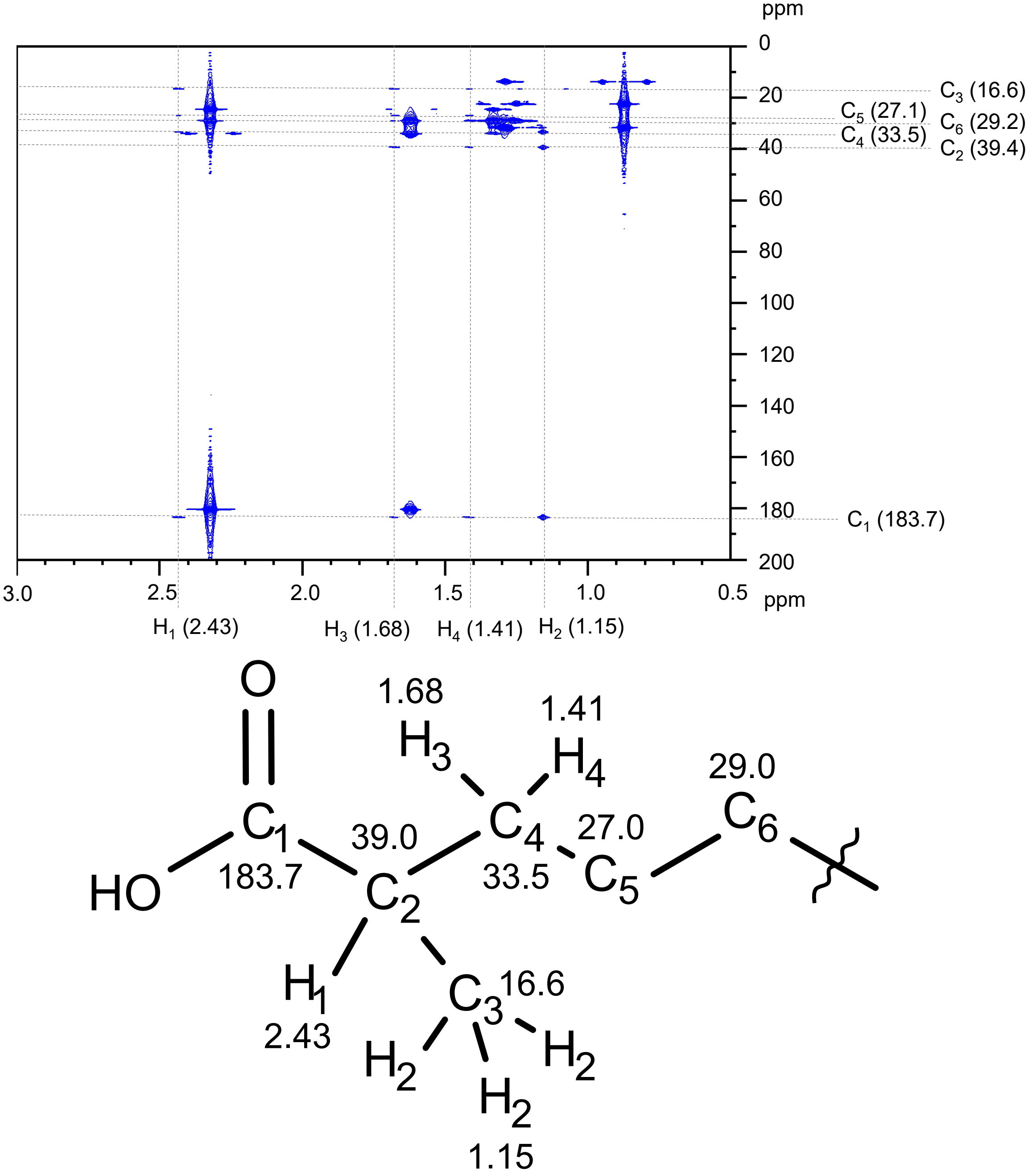

Fig. S13.
2D-NMR (^{1}H–^{13}C HMBC) spectrum of commercial nonanoic acid (TCI) before purification by recrystallization. The most intense impurity peak at 183.7 ppm (about 3% of the nonanoic acid peaks) originates from a carboxylic acid having a methyl (–CH_3) group at the α-position. Lower schematic illustration shows a possible structure of the most abundant impurity. H and C atoms are labeled with their chemical shifts from the ^{1}H–^{13}C HMBC spectrum.

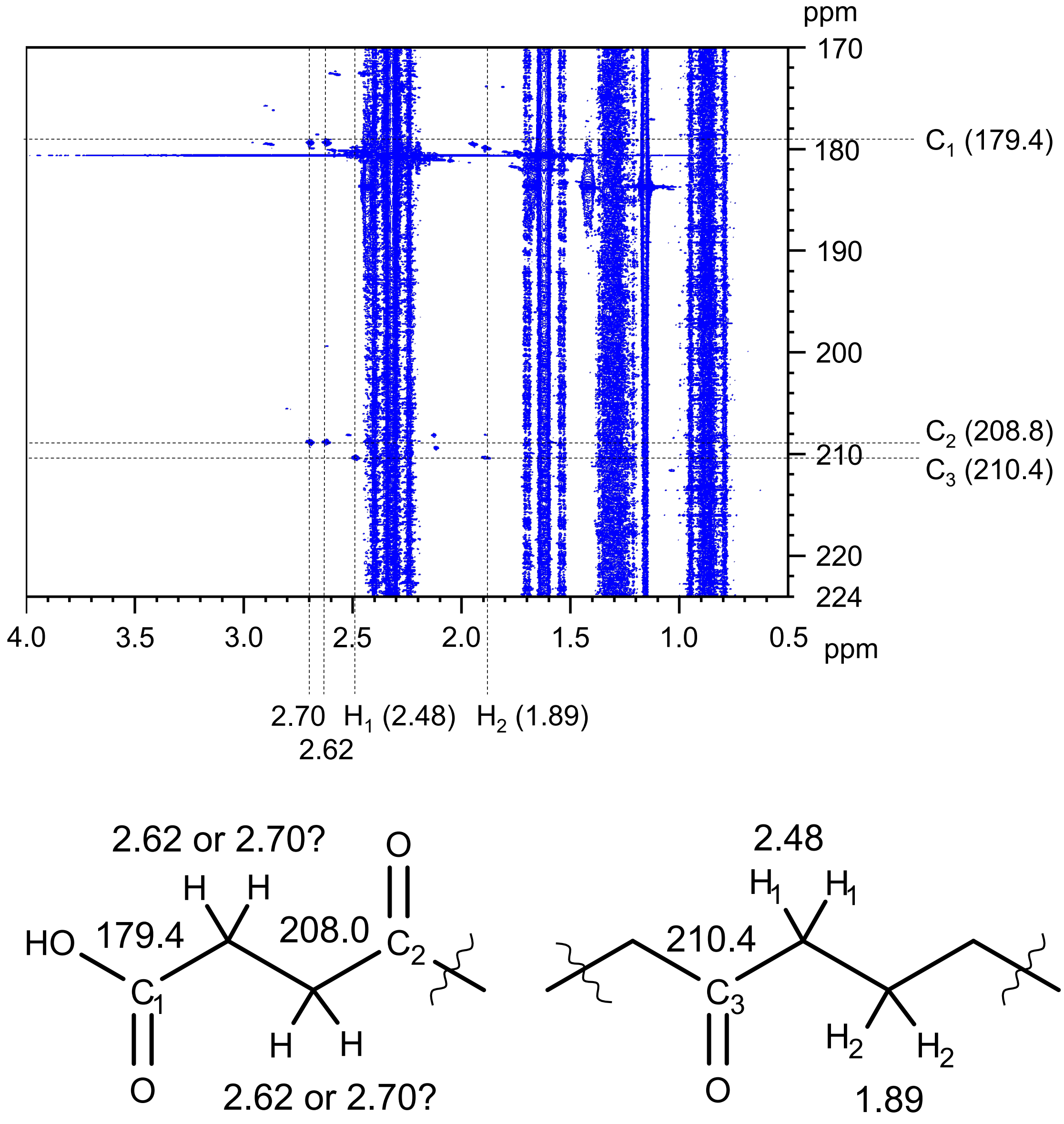

Fig. S14.

2D-NMR (^{1}H–^{13}C HMBC) spectrum of commercial nonanoic acid (TCI) before purification by recrystallization. The impurity peaks at 208.8 and 179.4 ppm originate from a keto acid. The impurity peak at 210.4 ppm was assigned to a ketone. Lower schematic illustrations show possible structures of the keto-acid and ketone impurities with the H and C atoms labeled with their chemical shifts from the ^{1}H–^{13}C HMBC spectrum.

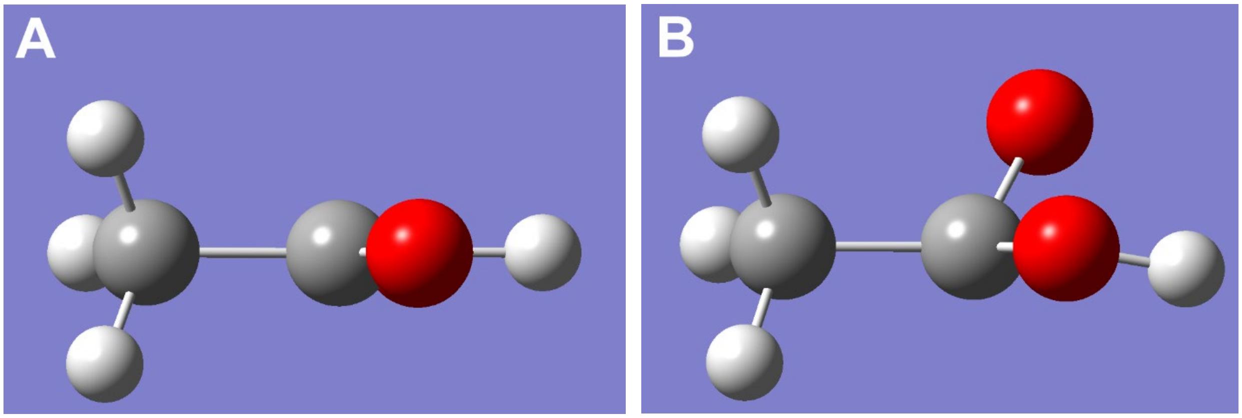

Fig. S15.

Optimized structures of acetic acid showing the (A) singlet (S_0) and (B) triplet (T_1) state. (A) shows a typical planar structure for the –COOH moiety, whereas (B) has a significantly changed conformation. Therefore, a triplet state in the (A) geometry is far from its relaxed conformation.

Table S1.
Summary of the conditions for measuring UV absorption spectra.

L (L')[a] (mm)	L^s (mm)	L^b (mm)	Method	Number of trials	Wavelength used for calculation of σ (nm)
0.010 (0.0185 ± 0.0002)	0.025	0.015	Liquid film	7	190–229
0.085 (0.105 ± 0.004)	0.1	0.015	Liquid film	6	227–238
0.5 (0.564 ± 0.014)	1.0	0.5	Liquid film	4	236–245
1	2	1	Cuvette	3	239–247
4	5	1	Cuvette	3	245–253
10	20	10	Cuvette	3	248–256
40	50	10	Cuvette	3	254–281
90	100	10	Cuvette	3	257–310

[a] L' represents the actual optical path length in the liquid film measurements. Errors indicate the statistical errors (1 sigma). For details, see the Material and Method section in the main text.

Table S2.

Absorption cross sections (*σ*) of commercial nonanoic acid (TCI) before and after purification by recrystallization.

Wavelength (λ, nm)	Absorption cross section before recrystallization (σ, cm^2)	Error (1 sigma) (cm^2)	Absorption cross section after recrystallization (σ, cm^2)	Error (1 sigma) (cm^2)
190	1.61E-19	0.03E-19	1.40E-19	0.03E-19
191	1.66E-19	0.02E-19	1.43E-19	0.02E-19
192	1.76E-19	0.02E-19	1.52E-19	0.02E-19
193	1.83E-19	0.02E-19	1.61E-19	0.02E-19
194	1.91E-19	0.02E-19	1.71E-19	0.02E-19
195	2.01E-19	0.02E-19	1.80E-19	0.02E-19
196	2.10E-19	0.01E-19	1.90E-19	0.03E-19
197	2.20E-19	0.02E-19	2.00E-19	0.03E-19
198	2.29E-19	0.02E-19	2.08E-19	0.03E-19
199	2.38E-19	0.01E-19	2.16E-19	0.03E-19
200	2.45E-19	0.01E-19	2.22E-19	0.03E-19
201	2.51E-19	0.01E-19	2.28E-19	0.03E-19
202	2.55E-19	0.02E-19	2.33E-19	0.03E-19
203	2.59E-19	0.02E-19	2.37E-19	0.03E-19
204	2.61E-19	0.01E-19	2.40E-19	0.03E-19
205	2.62E-19	0.01E-19	2.41E-19	0.03E-19
206	2.62E-19	0.01E-19	2.40E-19	0.03E-19
207	2.60E-19	0.01E-19	2.39E-19	0.03E-19
208	2.56E-19	0.01E-19	2.35E-19	0.03E-19
209	2.51E-19	0.01E-19	2.31E-19	0.03E-19
210	2.45E-19	0.01E-19	2.25E-19	0.03E-19
211	2.38E-19	0.01E-19	2.19E-19	0.02E-19
212	2.30E-19	0.01E-19	2.11E-19	0.02E-19
213	2.21E-19	< 0.01E-19	2.02E-19	0.02E-19
214	2.11E-19	< 0.01E-19	1.93E-19	0.02E-19
215	2.00E-19	< 0.01E-19	1.83E-19	0.02E-19
216	1.89E-19	0.01E-19	1.72E-19	0.02E-19
217	1.78E-19	0.01E-19	1.61E-19	0.01E-19
218	1.66E-19	0.01E-19	1.49E-19	0.01E-19

219	1.54E-19	0.01E-19	1.38E-19	0.01E-19
220	1.41E-19	0.01E-19	1.26E-19	0.01E-19
221	1.29E-19	< 0.01E-19	1.15E-19	0.01E-19
222	1.17E-19	0.01E-19	1.04E-19	0.01E-19
223	1.06E-19	< 0.01E-19	9.33E-20	0.05E-20
224	9.58E-20	0.05E-20	8.32E-20	0.05E-20
225	8.53E-20	0.07E-20	7.37E-20	0.03E-20
226	7.55E-20	0.07E-20	6.48E-20	0.03E-20
227	6.65E-20	0.06E-20	5.61E-20	0.04E-20
228	5.85E-20	0.02E-20	4.83E-20	0.03E-20
229	5.11E-20	0.01E-20	4.14E-20	0.03E-20
230	4.43E-20	0.01E-20	3.55E-20	0.03E-20
231	3.82E-20	0.01E-20	3.00E-20	0.02E-20
232	3.27E-20	0.02E-20	2.52E-20	0.02E-20
233	2.82E-20	0.01E-20	2.10E-20	0.02E-20
234	2.41E-20	0.01E-20	1.74E-20	0.01E-20
235	2.04E-20	0.01E-20	1.42E-20	0.01E-20
236	1.72E-20	< 0.01E-20	1.13E-20	0.03E-20
237	1.44E-20	0.01E-20	9.12E-21	0.18E-21
238	1.19E-20	0.01E-20	7.27E-21	0.19E-21
239	1.00E-20	< 0.01E-20	5.88E-21	0.20E-21
240	8.44E-21	0.05E-21	4.72E-21	0.16E-21
241	7.10E-21	0.07E-21	3.75E-21	0.12E-21
242	5.96E-21	0.07E-21	2.94E-21	0.10E-21
243	5.03E-21	0.04E-21	2.28E-21	0.08E-21
244	4.26E-21	0.02E-21	1.77E-21	0.06E-21
245	3.60E-21	0.01E-21	1.36E-21	0.04E-21
246	3.07E-21	0.01E-21	1.05E-21	0.02E-21
247	2.64E-21	0.01E-21	8.23E-22	0.17E-22
248	2.27E-21	0.01E-21	6.00E-22	0.44E-22
249	1.97E-21	0.01E-21	4.73E-22	0.29E-22
250	1.72E-21	0.01E-21	3.70E-22	0.20E-22
251	1.51E-21	0.01E-21	2.88E-22	0.14E-22
252	1.35E-21	0.01E-21	2.25E-22	0.09E-22
253	1.22E-21	0.01E-21	1.75E-22	0.06E-22
254	1.11E-21	0.01E-21	1.33E-22	0.03E-22

255	1.02E-21	0.01E-21	1.06E-22	0.03E-22
256	9.44E-22	0.05E-22	8.40E-23	0.36E-23
257	8.81E-22	0.06E-22	6.94E-23	0.18E-23
258	8.27E-22	0.04E-22	5.68E-23	0.17E-23
259	7.85E-22	0.05E-22	4.76E-23	0.17E-23
260	7.49E-22	0.03E-22	4.09E-23	0.16E-23
261	7.20E-22	0.04E-22	3.57E-23	0.16E-23
262	6.98E-22	0.03E-22	3.19E-23	0.16E-23
263	6.78E-22	0.04E-22	2.91E-23	0.17E-23
264	6.63E-22	0.01E-22	2.69E-23	0.17E-23
265	6.51E-22	0.01E-22	2.52E-23	0.17E-23
266	6.42E-22	0.01E-22	2.41E-23	0.17E-23
267	6.34E-22	0.01E-22	2.32E-23	0.17E-23
268	6.28E-22	0.01E-22	2.25E-23	0.16E-23
269	6.22E-22	0.01E-22	2.20E-23	0.16E-23
270	6.17E-22	0.01E-22	2.15E-23	0.16E-23
271	6.12E-22	0.01E-22	2.12E-23	0.16E-23
272	6.09E-22	0.01E-22	2.08E-23	0.17E-23
273	6.04E-22	0.01E-22	2.05E-23	0.17E-23
274	5.99E-22	0.01E-22	2.02E-23	0.17E-23
275	5.94E-22	0.01E-22	2.01E-23	0.17E-23
276	5.87E-22	0.01E-22	2.00E-23	0.16E-23
277	5.79E-22	0.01E-22	1.98E-23	0.16E-23
278	5.68E-22	0.02E-22	1.95E-23	0.16E-23
279	5.56E-22	0.01E-22	1.92E-23	0.16E-23
280	5.43E-22	0.01E-22	1.90E-23	0.16E-23
281	5.32E-22	0.01E-22	1.87E-23	0.16E-23
282	5.19E-22	0.01E-22	1.94E-23	0.13E-23
283	5.07E-22	0.01E-22	1.92E-23	0.12E-23
284	4.93E-22	0.01E-22	1.89E-23	0.12E-23
285	4.77E-22	0.01E-22	1.85E-23	0.11E-23
286	4.58E-22	0.01E-22	1.81E-23	0.11E-23
287	4.38E-22	0.01E-22	1.76E-23	0.10E-23
288	4.19E-22	0.01E-22	1.70E-23	0.10E-23
289	4.01E-22	0.01E-22	1.64E-23	0.10E-23
290	3.83E-22	0.01E-22	1.59E-23	0.09E-23

291	3.67E-22	0.01E-22	1.54E-23	0.09E-23
292	3.51E-22	< 0.01E-22	1.50E-23	0.09E-23
293	3.36E-22	< 0.01E-22	1.45E-23	0.09E-23
294	3.21E-22	0.01E-22	1.39E-23	0.09E-23
295	3.07E-22	0.01E-22	1.33E-23	0.09E-23
296	2.92E-22	0.01E-22	1.27E-23	0.08E-23
297	2.78E-22	0.01E-22	1.19E-23	0.08E-23
298	2.64E-22	< 0.01E-22	1.11E-23	0.08E-23
299	2.51E-22	0.01E-22	1.03E-23	0.08E-23
300	2.39E-22	0.01E-22	9.63E-24	0.76E-24
301	2.27E-22	< 0.01E-22	9.01E-24	0.72E-24
302	2.16E-22	< 0.01E-22	8.49E-24	0.72E-24
303	2.04E-22	0.01E-22	8.02E-24	0.68E-24
304	1.94E-22	0.01E-22	7.60E-24	0.72E-24
305	1.84E-22	0.01E-22	7.30E-24	0.72E-24
306	1.74E-22	0.01E-22	7.03E-24	0.69E-24
307	1.66E-22	0.01E-22	6.78E-24	0.65E-24
308	1.58E-22	0.01E-22	6.54E-24	0.66E-24
309	1.50E-22	0.01E-22	6.39E-24	0.66E-24
310	1.42E-22	< 0.01E-22	6.24E-24	0.66E-24

Table S3.

Absorption cross sections (*σ*) and photolysis quantum yields (*Φ*) of formaldehyde (H_2CO).[a]

Wavelength (λ, nm)	Absorption cross section[b] (σ, cm^2)	Quantum yield Φ(H + HCO)[c]	Quantum yield Φ(H_2 + CO)[d]	Φ(H + HCO) + Φ(H_2 + CO)[e]
292	8.60E-21	0.710	0.266	0.98
293	3.36E-20	0.718	0.262	0.98
294	7.72E-20	0.726	0.259	0.99
295	4.38E-20	0.734	0.256	0.99
296	2.68E-20	0.74	0.254	0.99
297	1.47E-20	0.746	0.252	1.00
298	4.56E-20	0.751	0.249	1.00
299	3.42E-20	0.755	0.245	1.00
300	1.04E-20	0.758	0.242	1.00
301	1.76E-20	0.761	0.239	1.00
302	9.20E-21	0.762	0.238	1.00
303	3.26E-20	0.762	0.238	1.00
304	7.81E-20	0.762	0.238	1.00
305	5.12E-20	0.76	0.24	1.00
306	4.63E-20	0.758	0.242	1.00
307	1.92E-20	0.754	0.246	1.00
308	1.49E-20	0.749	0.251	1.00
309	3.52E-20	0.744	0.256	1.00
310	1.88E-20	0.737	0.263	1.00
311	4.98E-21	0.729	0.271	1.00
312	1.28E-20	0.72	0.28	1.00
313	9.74E-21	0.709	0.291	1.00
314	6.10E-20	0.698	0.302	1.00
315	6.00E-20	0.685	0.315	1.00
316	2.74E-20	0.671	0.329	1.00
317	6.25E-20	0.656	0.344	1.00
318	3.40E-20	0.639	0.361	1.00
319	1.05E-20	0.622	0.378	1.00
320	1.28E-20	0.603	0.397	1.00
321	1.73E-20	0.583	0.417	1.00
322	7.79E-21	0.561	0.439	1.00

323	3.53E-21	0.539	0.461	1.00
324	9.30E-21	0.515	0.485	1.00
325	1.66E-20	0.489	0.511	1.00
326	7.42E-20	0.463	0.537	1.00
327	4.72E-20	0.435	0.565	1.00
328	1.32E-20	0.406	0.594	1.00
329	3.37E-20	0.375	0.625	1.00
330	4.17E-20	0.343	0.657	1.00
331	1.52E-20	0.31	0.69	1.00
332	3.74E-21	0.276	0.714	0.99
333	2.31E-21	0.24	0.73	0.97
334	1.72E-21	0.203	0.737	0.94
335	1.04E-21	0.165	0.735	0.90
336	1.36E-21	0.126	0.724	0.85
337	4.14E-21	0.085	0.705	0.79
338	2.07E-20	0.043	0.687	0.73
339	5.94E-20	0	0.665	0.67
340	3.40E-20	–	0.645	0.65
341	1.06E-20	–	0.62	0.62
342	5.44E-21	–	0.59	0.59
343	2.07E-20	–	0.56	0.56
344	1.37E-20	–	0.53	0.53
345	4.71E-21	–	0.505	0.51
346	1.28E-21	–	0.48	0.48
347	4.76E-22	–	0.45	0.45
348	8.18E-22	–	0.425	0.43
349	4.08E-22	–	0.4	0.40
350	3.89E-22	–	0.375	0.38
351	9.66E-22	–	0.35	0.35
352	7.90E-21	–	0.32	0.32
353	2.46E-20	–	0.285	0.29
354	1.78E-20	–	0.25	0.25
355	7.52E-21	–	0.22	0.22
356	1.60E-21	–	0.19	0.19
357	3.72E-22	–	0.16	0.16
358	2.01E-22	–	0.13	0.13

359	1.20E-22	–	0.09	0.09
360	9.40E-23	–	0.04	0.04
361	1.08E-22	–	0	0.00

[a] Recommended values reported in reference (*3*).
[b] Averaged values over 1 nm intervals at 298 K are shown because the UV absorption spectrum of formaldehyde (H_2CO) displays a highly structured absorption band between 240 and 380 nm. Reference (*3*) gives details.
[c] Quantum yields of $H_2CO \rightarrow H + HCO$ at 300 K and atmospheric pressure.
[d] Quantum yields of $H_2CO \rightarrow H_2 + CO$ at 300 K and atmospheric pressure.
[e] Values used for calculating the photolysis rate in the main text.

Table S4.

Absorption cross sections (σ) and photolysis quantum yields (Φ) of acetaldehyde (CH_3CHO).[a]

Wavelength (λ, nm)	Absorption cross section[b] (σ, cm^2)	Quantum yield $\Phi(CH_3 + HCO)$[c]	Quantum yield $\Phi(CH_4 + CO)$[d]	$\Phi(CH_3 + HCO)$ + $\Phi(CH_4 + CO)$[e]
292	4.66E-20	0.52	0.005	0.525
293	4.51E-20	–	–	–
294	4.31E-20	0.50	0	0.50
295	4.26E-20	–	–	–
296	4.24E-20	0.47	–	0.47
297	4.37E-20	–	–	–
298	4.41E-20	0.45	–	0.45
299	4.26E-20	–	–	–
300	4.15E-20	0.43	–	0.43
301	3.97E-20	–	–	–
302	3.87E-20	0.40	–	0.40
303	3.70E-20	–	–	–
304	3.46E-20	0.38	–	0.38
305	3.43E-20	–	–	–
306	3.41E-20	0.35	–	0.35
307	3.36E-20	–	–	–
308	3.31E-20	0.31	–	0.31
309	3.11E-20	–	–	–
310	2.92E-20	0.28	–	0.28
311	2.73E-20	–	–	–
312	2.52E-20	0.24	–	0.24
313	2.47E-20	–	–	–
314	2.38E-20	0.19	–	0.19
315	2.20E-20	–	–	–
316	2.07E-20	0.15	–	0.15
317	2.08E-20	–	–	–
318	1.98E-20	0.12	–	0.12
319	1.84E-20	–	–	–
320	1.70E-20	0.10	–	0.10
321	1.48E-20	–	–	–

322	1.38E-20	0.07	–	0.07
323	1.23E-20	–	–	–
324	1.06E-20	0.05	–	0.05
325	1.15E-20	–	–	–
326	1.09E-20	0.03	–	0.03
327	8.08E-21	–	–	–
328	7.15E-21	0.02	–	0.02
329	7.41E-21	–	–	–
330	6.99E-21	0.01	–	0.01
331	5.60E-21	–	–	–
332	4.96E-21	0	–	0

[a] Recommended values reported in reference (*3*).
[b] 1 nm averages at 298 K. Reference (*3*) gives details.
[c] Quantum yields of $CH_3CHO \rightarrow CH_3 + HCO$ at room temperature and atmospheric pressure.
[d] Quantum yields of $CH_3CHO \rightarrow CH_4 + CO$ at room temperature and atmospheric pressure.
[e] Values used for calculating the photolysis rate in the main text. The integration is carried out at 292–332 nm using wavelengths that have photolysis quantum yields.

Table S5.

Absorption cross sections (σ) and photolysis quantum yields (Φ) of acetone ($CH_3C(O)CH_3$).[a]

Wavelength (λ, nm)	Absorption cross section[b] (σ, cm^2)	Quantum yield[c] Φ
292	3.82E-20	0.377
293	3.71E-20	0.361
294	3.57E-20	0.345
295	3.42E-20	0.330
296	3.26E-20	0.315
297	3.11E-20	0.301
298	2.98E-20	0.287
299	2.82E-20	0.274
300	2.67E-20	0.261
301	2.58E-20	0.249
302	2.45E-20	0.237
303	2.30E-20	0.213
304	2.18E-20	0.184
305	2.05E-20	0.159
306	1.89E-20	0.137
307	1.75E-20	0.119
308	1.61E-20	0.103
309	1.49E-20	0.0887
310	1.36E-20	0.0769
311	1.24E-20	0.0669
312	1.14E-20	0.0584
313	1.06E-20	0.0511
314	9.44E-21	0.0449
315	8.37E-21	0.0396
316	7.60E-21	0.0350
317	6.84E-21	0.0311
318	5.98E-21	0.0278
319	5.23E-21	0.0248
320	4.55E-21	0.0223
321	4.11E-21	0.0201
322	3.48E-21	0.0181

323	2.94E-21	0.0164
324	2.48E-21	0.0149
325	2.10E-21	0.0135
326	1.74E-21	0.0124
327	1.41E-21	0.0113

[a] Recommended values reported in reference (*3*).
[b] 1 nm averages at 298 K. Reference (*42*) reports the original data.
[c] Photolysis quantum yields at 295 K. References (*3*, *44)* give details. These values are used for calculating the photolysis rate in the main text.

Table S6.
Photon excitation of carboxylic acids by vertical transition.[a]

Entry	System	Method	Wavelength (nm)	
			Singlet (S_1)	Triplet (T_1)
S1	Nonanoic acid	TDDFT	209	231
S2	Propionic acid	TDDFT	208	231
S3	Acetic acid	TDDFT	210	232
S4	Acetic acid	DFT	-	233
S5	Acetic acid	SAC-CI	218	223
S6	Acetic acid dimer	TDDFT	202	220
S7	Acetic acid[b]	TDDFT	206	227
S8	Acetic acid dimer + H^+	TDDFT	196	215
S9	Acetic acid dimer - H^+	TDDFT	216	230
S10	Acetic acid $3H_2O$	TDDFT	201	220
S11	Acetic acid[c]	TDDFT	205	226

[a] TDDFT: CAM-B3LYP/6-311+G(2df,2p). DFT: B3LYP/6-311+G(2df,2p). SAC-CI: aug-cc-pVTZ. The wavelength corresponds to the energy needed for excitation from the ground state.
[b] IEFPCM incorporated the solvent effect of acetic acid.
[c] IEFPCM incorporated the solvent effect of water.

Table S7.
Actinic flux (photon cm^{-2} nm^{-1} s^{-1}) at 0.1 km altitude.[a]

Wavelength (λ, nm)	Actinic flux (photon cm^{-2} nm^{-1} s^{-1})
292	2.3987E9
293	6.4996E9
294	1.6562E10
295	5.1697E10
296	1.1973E11
297	2.8058E11
298	4.8606E11
299	9.2239E11
300	1.4375E12
301	2.4352E12
302	3.9961E12
303	7.1130E12
304	1.0038E13
305	1.3453E13
306	1.6268E13
307	2.1952E13
308	2.7521E13
309	2.8059E13
310	3.5464E13
311	4.8146E13
312	5.4028E13
313	5.9672E13
314	6.4548E13
315	6.7759E13
316	7.0507E13
317	8.6148E13
318	8.9124E13
319	9.0385E13
320	1.0730E14
321	1.0811E14
322	1.0235E14

323	1.0376E14
324	1.1774E14
325	1.3476E14
326	1.5941E14
327	1.6759E14
328	1.5942E14
329	1.7984E14
330	1.8747E14
331	1.7719E14
332	1.7731E14
333	1.738E14
334	1.7786E14
335	1.8590E14
336	1.6819E14
337	1.5954E14
338	1.7306E14
339	1.8443E14
340	1.9629E14
341	1.9017E14
342	1.9325E14
343	1.9754E14
344	1.6864E14
345	1.7843E14
346	1.9082E14
347	1.8587E14
348	1.8843E14
349	1.8366E14
350	2.0756E14
351	2.1292E14
352	1.9460E14
353	2.1110E14
354	2.3555E14
355	2.3260E14
356	2.1122E14
357	1.8928E14
358	1.5908E14

359	1.9726E14
360	2.2337E14
361	2.0548E14

[a] Data adopted from reference (*23*). Airborne measurements were made over the Aegean Sea on 10 June 1996 under cloud-free conditions. The solar zenith angle was 17°.
